# Minimizing Endothelial Cell Loss in Hard Nucleus Cataract Surgery: Efficacy of the Eight-Chop Technique

**DOI:** 10.3390/jcm14082576

**Published:** 2025-04-09

**Authors:** Tsuyoshi Sato

**Affiliations:** Department of Ophthalmology, Sato Eye Clinic, 3-3 Nemoto, Matsudo-shi 271-0077, Chiba-ken, Japan; perfect-eightchop@sato-ganka.com

**Keywords:** corneal endothelial cell, eight-chop technique, hard nucleus cataracts, phacoemulsification

## Abstract

**Objectives**: To estimate the efficacy of the eight-chop technique in phacoemulsification surgeries for patients with hard nucleus cataracts by investigating the reduction of corneal endothelial cell density (CECD) after phacoemulsification and intraoperative parameters. **Methods**: Patients were categorized into three groups (Grade IV, IV plus, and V) according to the hardness of their lens nuclei. Surgeries were performed using the eight-chop technique. Key intraoperative metrics (operative time, phaco time, aspiration time, cumulative dissipated energy [CDE], and fluid volume used) were measured. Pre- and postoperative assessments included corrected-distance visual acuity, intraocular pressure (IOP), central corneal thickness, variation in the size of the endothelial cells, percentage of hexagonal cells, and CECD. **Results**: Overall, 89 eyes from 67 patients with cataracts were evaluated. The mean operative time, phaco time, aspiration time, CDE, and fluid volume used across Grades IV, IV plus, and V were 10.5 min, 38.9 s, 135.6 s, 19.2, and 53.0 mL, respectively. At 19 weeks postoperatively, the CECD decreased by 0.2%, 6.8%, and 9.6% for Grades IV, IV plus, and V, respectively, with an average decrease of 3.7%. Significant reductions in postoperative IOP were observed across all groups compared with preoperative IOP (*p* < 0.01). Loss of CECD significantly correlated with phaco time, CDE, and fluid volume (*p* = 0.027, *p* < 0.01, and 0.034, respectively). **Conclusions**: The eight-chop technique in phacoemulsification for hard nucleus cataracts resulted in minimal CECD loss. It may provide an effective surgical solution for patients with hard nucleus cataracts.

## 1. Introduction

Cataract surgery in patients with a hard nucleus presents significant challenges, even for the most experienced surgeons, often leading to complications. Splitting the dense nucleus using conventional techniques is particularly challenging. Hard nucleus cataracts represent the most advanced stage of cataract formation, where the nuclei have achieved maximum density [1]. Many variations of phacoemulsification techniques have been developed to overcome hard nucleus cataracts [2,3,4,5,6,7], although many problems remain in terms of complications, particularly corneal endothelial cell density (CECD) loss. Although femtosecond laser-assisted cataract surgery is considered advantageous in terms of reducing the use of ultrasonic oscillation energy, CECD reduction rates of 7.9% to 11.7% have been reported, which is comparable to that of conventional cataract surgery [7,8]. The idea of femtosecond laser prefragmentation is also used with the eight-chop technique, where the lens nucleus is divided without the use of ultrasonic oscillation energy.

The eight-chop technique involves dividing the lens nucleus into eight sections before phacoemulsification and aspiration. It is more effective than other techniques for particularly hard nucleus cataracts. First, by supporting the equatorial part of the nucleus with an instrument, the mechanical impact on the zonules and posterior lens capsule can be minimized. Second, the lens nucleus can be safely divided in the stable anterior chamber filled with an ophthalmic viscosurgical device. Third, the eight-chopper has a sharp tip and can therefore divide almost all hard lens nuclei. This technique allows for efficient processing of the lens nucleus, reducing operative time, cumulative dissipated energy (CDE), and fluid volume while minimizing corneal endothelial cell loss [9,10]. A study reported a decrease in CECD of 0.9%, 1.0%, and 5.3% in the Grade II, III, and IV groups, respectively, and an operative time of 3.7 min, 5.4 min, and 9.6 min in the Grade II, III, and IV groups, respectively [9]. Additionally, it may be particularly effective for patients with hard nucleus cataracts during phacoemulsification cataract surgery. Therefore, it is clinically meaningful to examine hard nucleus cataracts in detail, including Grade V cases to Grade IV cases.

Several studies have identified preoperative and intraoperative factors that influence the risk of endothelial cell loss after phacoemulsification, such as hard nucleus density, high ultrasound energy, prolonged phacoemulsification time, the specific phacoemulsification technique employed, and the use of large infusion volumes to increase the risk of endothelial cell loss following phacoemulsification [5,11,12,13,14,15]. However, these studies did not consider surgical factors, such as operative time, phaco time, CDE, aspiration time, and fluid volume used, as confounding factors. Thus, a thorough evaluation of intraoperative parameters and their impact on CECD reduction after phacoemulsification is needed.

Therefore, in this study, we investigated the intraoperative parameters and CECD reduction in patients with hard nucleus cataracts who underwent phacoemulsification cataract surgery using the eight-chop technique.

## 2. Materials and Methods

### 2.1. Ethical Considerations

The study protocol adhered to the guidelines of the Declaration of Helsinki and was approved by the Sato Eye Clinic review board (approval number 20150401). Informed consent for participation in this study was obtained from each patient after a thorough explanation of the study’s nature and potential consequences.

### 2.2. Study Population

The study consecutively recruited Japanese patients who had phacoemulsification for cataracts using the eight-chop technique with implantation of a posterior chamber intraocular lens (IOL). Between September 2015 and August 2023, 5959 cataract surgeries were performed, of which 160 eyes had a Grade IV or V nucleus. Among these, 89 eyes met the inclusion criteria and were evaluated postoperatively. Patients with cataracts who underwent phacoemulsification and IOL implantation in the posterior chamber were included in the study. Exclusion criteria were corneal disease or opacity, uveitis, poorly dilated pupils (<5.0 mm), all-white cortical cataracts, preoperative CECD < 2000 cells/mm^2^, severe weak zonules, and previous ocular trauma or surgery.

### 2.3. Preoperative Assessment

**Figure 1:**
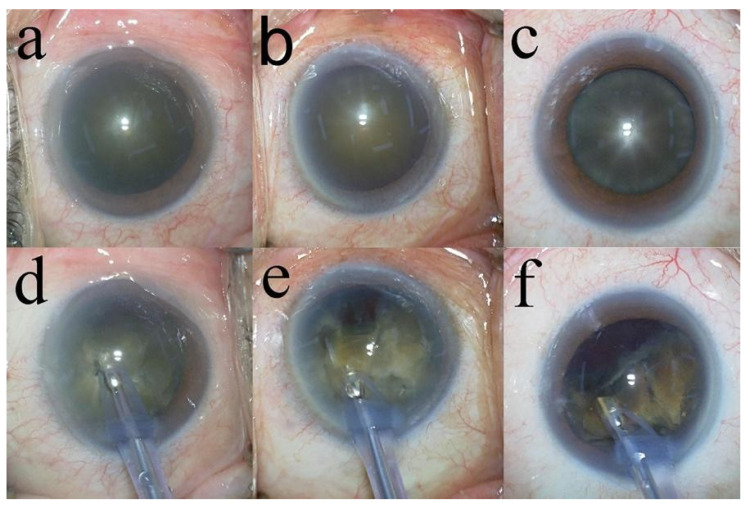
Grade IV, IV plus, and V cataracts. Frontal views of Grade IV nucleus (**a**), Grade IV plus nucleus (**b**), and Grade V nucleus (**c**). Grade IV nucleus, completely amber (**d**). Grade IV plus nucleus, mostly amber but some brown (**e**). Grade V nucleus, mostly brown but some black (**f**).

Preoperatively, all patients underwent slit-lamp and retinal examinations, and their corrected distance visual acuity (CDVA) and intraocular pressure (IOP) were measured. The CECD (cells/mm^2^), central corneal thickness (CCT), variation in the size of the endothelial cells (CV), and percentage of hexagonal cells (PHC) were analyzed using a non-contact specular microscope (EM-3000; Topcon Corporation, Tokyo, Japan). Cataracts were graded according to the Emery–Little classification [16] using a slit lamp. Patients with Grade IV or V nuclei, defined as hard nucleus cataracts, were included in the study. Additionally, cases classified as Grade IV but with a partially brown posterior plate during surgery were classified as Grade IV plus (Figure 1).

### 2.4. Surgical Technique

**Figure 2:**
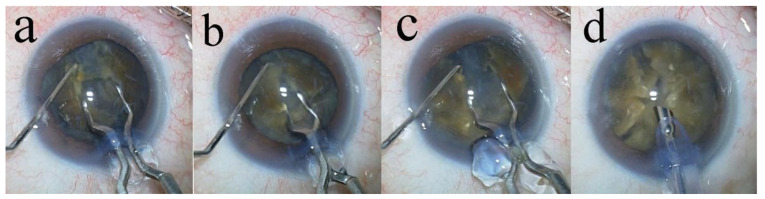
Lens nucleus segmentation of Grade V cataract. (**a**) First, the lens nucleus is divided into hemispheres using the Lance-chopper. (**b**) Thereafter, a 90° rotation of the lens nucleus is performed, and it is divided into quadrants. (**c**) The lens nucleus that has been divided into quadrants is rotated again at a 45° angle and then divided into six segments. (**d**) Lastly, the remaining quadrants of the lens nucleus are also divided to complete the eight-part segmentation.

The same surgeon, experienced in the eight-chop technique, performed phacoemulsification using the phacoemulsification unit (Centurion^®^; Alcon Laboratories, Inc., Irvine, CA, USA) in all patients. To facilitate the eight-chop technique, new surgical instruments have been specifically designed and developed [9]. Among these is the Lance-chopper (SP-9989; ASICO, Parsippany, NJ, USA), which features a smaller tip compared to the Universal II Prechopper (AE-4192; ASICO, Parsippany, NJ, USA), with a length and width of 3.0 mm and 1.2 mm, respectively, and a sharper leading edge. In all surgeries, a temporal, clear corneal incision was made using a 3.0 mm steel keratome. Brilliant blue G (0.025%) was applied to enhance capsule visualization in all cases. After injecting sodium hyaluronate into the anterior chamber, a continuous curvilinear capsulorhexis, measuring 6.2–6.5 mm, was created using a capsule forceps. The soft-shell technique was used in all cases [17]. Hydrodissection was performed using a 27-G cannula; however, hydrodelineation was not performed. The lens nucleus was divided into eight segments using a Lance-chopper (Figure 2), and these segments were phacoemulsified and aspirated at the depth of the iris plane. The capsular bag was aspirated with an irrigation/aspiration tip to remove residual cortical materials. An ophthalmic viscosurgical device was injected, and a foldable IOL (Acrysof^®^ MN60AC; Alcon Laboratories, Inc., Fort Worth, TX, USA) with polymethyl methacrylate haptics was inserted into the capsular bag using an injector system. Subsequently, the ophthalmic viscosurgical device was aspirated. A phacoemulsification unit was used in all cases, with a flow rate of 32 mL/min, maximum ultrasound power of 80%, and a 1.1-mm tip. If necessary, the wound was sealed using stromal hydration. The anterior chamber was then filled with a balanced salt solution containing moxifloxacin (0.5 mg/mL) for postoperative care. All surgeries were recorded using a camera (MKC-704KHD, Ikegami Tsushinki Co., Ltd., Tokyo, Japan), and the video footage was stored on a hard disk drive.

### 2.5. Measures

Intraoperative outcome measures included operative time (min), phaco time (s), aspiration time (s), CDE, fluid volume used (mL), and rate of intraoperative complications. Operative time was defined as the duration from the initiation of the corneal incision to the completion of ophthalmic viscosurgical device aspiration.

### 2.6. Data Collection

Patients were followed up on postoperative days 1 and 2 and on postoperative weeks 1, 3, 7, and 19. The postoperative outcome measures included CDVA, IOP, CCT, CV, PHC, and CECD obtained at 7 and 19 weeks postoperatively. Since the study focused on examining changes in CECD and IOP, cases in which accurate CECD and IOP could not be measured during the pre- and postoperative observation periods were excluded.

### 2.7. Statistical Analysis

The chi-square test was used to compare sex distribution among the groups (Grades IV, IV plus, and V). One-way analysis of variance was employed to compare results among groups. Paired *t*-tests were used to compare the preoperative values of CDVA, IOP, CV, PHC, CCT, and CECD with those at each postoperative time point. Statistical significance was set at *p* < 0.05. All statistical analyses were performed using Excel-Toukei (v. 7.0, Esumi Co. Ltd., Tokyo, Japan).

## 3. Results

### 3.1. Participant Characteristics

This study included 89 eyes from 67 patients with cataracts who underwent phacoemulsification and posterior chamber IOL implantation. Table 1 presents the patient characteristics and intraoperative parameters. No significant differences were observed in the mean age (*p* = 0.62) or sex (*p* = 0.55) in the Grade IV, IV plus, and V groups. However, significant differences were observed in the operative time, phaco time, aspiration time, CDE, and volume of fluid used in the Grade IV, IV plus, and V groups (all *p* < 0.01).

### 3.2. Changes in CECD

Table 2 shows the preoperative and postoperative changes in CECD. No significant differences in CECD were observed preoperatively or at 19 weeks postoperatively among the Grade IV, IV plus, and V groups (*p* = 0.35 and 0.09, respectively). However, significant differences in CECD were observed at 7 weeks postoperatively among the groups (*p* = 0.01). Within the Grade IV group, no significant differences were found between preoperative CECD and that at 7 weeks (*p* = 0.42) or 19 weeks (*p* = 0.29) postoperatively. However, significant differences were observed between preoperative CECD and that at 7 and 19 weeks postoperatively in the Grade IV plus (*p* < 0.01, and *p* = 0.02, respectively) and V groups (both *p* = 0.04).

### 3.3. Changes in CCT, CV, and PHC

Table 3 presents the preoperative and postoperative changes in CCT, CV, and PHC. No significant differences were observed between CCT preoperatively and at 7 or 19 weeks postoperatively among the Grade IV, IV plus, and V groups (*p* = 0.33, 0.50, and 0.55, respectively). Significant differences in CV were found preoperatively and at 7 weeks postoperatively among the Grade IV, IV plus, and V groups (all *p* < 0.01). However, no significant differences were observed between preoperative CV and that at 19 weeks postoperatively among the Grade IV, IV plus, and V groups (*p* = 0.06). Significant differences were observed in PHC preoperatively and at 7 weeks postoperatively among the Grade IV, IV plus, and V groups (*p* < 0.01, and *p* = 0.04, respectively) but not at 19 weeks postoperatively (*p* = 0.13).

### 3.4. Changes in IOP

Table 4 shows the changes in IOP. No significant differences were observed between preoperative IOP and that at 7 and 19 weeks postoperatively among the Grade IV, IV plus, and V groups (*p* = 0.84, 0.078, and 0.79, respectively). Significant differences were observed between preoperative IOP and that at 7 weeks postoperatively among the Grade IV, IV plus and V groups (all *p* < 0.01). However, significant differences were observed between preoperative IOP and that at 19 weeks postoperatively among the Grade IV and IV plus groups (all *p* < 0.01) but not in the Grade V group (*p* = 0.09).

### 3.5. Changes in CDVA over Time

Table 5 shows the changes in CDVA. No significant differences were observed in preoperative CDVA or that at 7 and 19 weeks postoperatively among the Grade IV, IV plus, and V groups (*p* = 0.07, 0.86, and 0.93, respectively). Significant differences were observed between preoperative CDVA and that at 7 and 19 weeks postoperatively among the Grade IV, IV plus, and V groups (all *p* < 0.01).

### 3.6. Correlation Between Loss of CECD and Surgical Parameters and IOP and Surgical Parameters

Loss of CECD correlated significantly with phaco time, CDE, and volume of fluid used (*p* = 0.027, *p* < 0.01, and 0.034, respectively). However, no significant correlations were observed between IOP and other surgical parameters.

### 3.7. Complications

Intraocular complications in 160 eyes with a Grade IV or V nucleus included a posterior capsule rupture in 2 eyes (1.3%) but did not include dialysis of the lens zonules, nuclear drop, or capsulorhexis tear. Two cases with complications were excluded from the analysis.

## 4. Discussion

The treatment of hard nucleus cataracts remains a significant challenge globally. In this study, we investigated the effect of the eight-chop technique for phacoemulsification on CECD and intraoperative parameters in patients with hard nucleus cataracts. The results demonstrated that using the eight-chop technique led to a minimal decrease in CECD after phacoemulsification and produced excellent intraoperative parameters, suggesting that the eight-chop technique could provide an effective approach for the removal of hard nucleus cataracts in many patients.

The complete division of a hard nucleus cataract can be particularly difficult with conventional techniques. This is because the radial suture plane of these lenses tends to be very adhesive around the posterior epinucleus, forming a dense posterior nuclear plate [18,19,20,21]. Surgeons often attempt to complete the division by moving the instruments and the fragments outward to extend the centrifugal traction, although this may inadvertently lead to excessive capsular bag distortion and stress to the zonular fibers [21]. When treating a hard nucleus, higher ultrasound power and longer phacoemulsification times are often required. Damage to the corneal incision, corneal endothelium, iris, and other intraocular tissue, and even bullous keratopathy and other serious surgery complications may occur due to mechanical trauma from sonic waves and thermal injury [13]. In previous studies, endothelial cell loss ranged from 4.3% to 37.0% in hard nucleus cases [5,6,7,8,14,15,22,23]. Corneal endothelial cells are non-replicative, and the loss of these cells is only compensated for by cell migration, enlargement, and increasing heterogeneity [24]. Moreover, cataract surgery has been reported to be the most common cause of bullous keratopathy [25].

Various phacoemulsification techniques have been developed over time [2,4]. Phacoemulsification began with the single-handed engraving technique and evolved into the divide-and-conquer technique, introduced by Gimbel in 1991 [26], followed by the phaco-chop technique reported by Nagahara in 1993 [27], and the prechop technique described by Akahoshi in 1994 [28]. As the use of ultrasonic oscillation energy increases the risk of corneal endothelial cell injury [29], all these techniques aim to reduce the total ultrasound time and energy used during nucleus emulsification.

The eight-chop technique involves dividing the lens nucleus into eight segments, compared to only four segments in the prechop technique [9,10]. When the eight-chopper cannot be inserted into a hard lens, the Lance-chopper is used to divide the lens nucleus by inserting it into the lens nucleus while supporting the lens equator with the nucleus sustainer, thereby minimizing stress on the zonules [9,10]. If the eight-chop technique reduces CECD loss and improves intraoperative parameters compared with previous techniques, this approach may be valuable for hard nucleus cataract surgery.

In this study, the operative time was 10.5 min, which is comparable to the operative times of regular cataract surgeries using other techniques [17,30]. While few studies have reported the operative times for hard nucleus cataract surgery (12.1 min for the drill-and-crack technique and 12.3 min for the phaco-chop technique) [6], none have documented shorter operative times than that observed in this study. Our results represent the shortest operative time reported to date, despite several existing reports on phaco time [8,23]. The aspiration time for cataract surgery for a hard lens nucleus has not been previously reported, although the aspiration time for normal cataract surgery has been documented as 244.7 s [31]. In contrast, this study found an aspiration time of 135.6 s, which is notably short, considering the hardness of the lens nucleus. Regarding CDE, our results align with those from previous studies [15,23]. However, when phacoemulsification is performed efficiently, CDE primarily depends on the hardness of the lens nucleus; thus, the technique’s superiority cannot be assessed solely on CDE values.

The volume of fluid used in this study was 53.0 mL, which is significantly lower than that typically reported for other techniques in standard cataract cases [12,32]. Previous studies have reported fluid volumes ranging from 105.9 to 221.7 mL for phacoemulsification of hard cataracts [33].

Previous studies have reported a 4.3–37.0% decrease in CECD following hard nucleus cataract surgery within the first few postoperative months [6,7,8,15,23]. However, in this study, at 19 weeks postoperatively, the decrease in CECD was slightly higher in the Grade IV plus and V groups (6.8% and 9.6%, respectively) than in the Grade IV group, which exhibited a minor decrease of 0.2%. The overall decrease was 3.7%, which is smaller than that previously reported [5,6,7,14,15,22]. These findings indicate that the eight-chop technique may be beneficial for minimizing CECD loss in hard nucleus cataract surgery.

The CCT is used as a marker of corneal endothelial function [34]. In this study, no significant differences in the preoperative and postoperative CCT were found among the Grade IV, IV plus, and V groups, indicating that corneal endothelial cell function remained normal postoperatively in all groups.

The CV measures the uniformity of endothelial cell size, reflecting the repair and healing mechanisms of the endothelium after damage. In this study, significant differences in CV were observed preoperatively, although no significant differences were observed in CV at 19 weeks postoperatively among the Grade IV, IV plus, and V groups. Additionally, postoperative CV was reduced; however, whether the repair function of corneal endothelial cells was activated remains unclear. Hexagonality indicates the variability in hexagonal cell shape, such as the CV, and represents the healing response after damage [35]. In this study, significant differences were observed between preoperative PHC and that at 7 weeks postoperatively, although no significant differences were noted between preoperative PHC and that at 19 weeks postoperatively among the Grade IV, IV plus, and V groups. This may suggest that the healing response of corneal endothelial cells in the Grade V group was reduced compared to those of the other groups at 7 weeks postoperatively but may have recovered at 19 weeks postoperatively.

Significant differences were observed between preoperative IOP and that at 7 and 19 weeks postoperatively in the overall cohort. The IOP reduction rates were 13.9% and 8.5% in the Grade IV and V groups, respectively, at 19 weeks postoperatively, indicating that a reduction in postoperative IOP can be expected even with hard nucleus cataracts.

The present study has some limitations. The first stems from the absence of the results with the prechop, phaco-chop, or divide-and-conquer techniques, and this should be fully considered when evaluating the present results. Second, it was difficult to include more patients with hard nucleus cataracts in this study because of the low morbidity of cataract patients with Grade IV and V lens nuclear hardness. Ideally, all ophthalmologists should be able to treat patients with hard nucleus cataracts, although given the complications, treatment should be left to specialists. In addition, if the lens is very hard, it can be treated with small-incision cataract surgery rather than phacoemulsification; however, in cases such as post-glaucoma surgery, phacoemulsification is necessary, and the surgical results of the eight-chop technique for hard nucleus cataracts require further study.

## 5. Conclusions

The eight-chop technique resulted in minimal CECD loss after phacoemulsification for hard nucleus cataracts. The intraoperative parameters also demonstrated excellent values, suggesting that the eight-chop technique may be an effective approach for hard nucleus cataract surgery. Recently, small-incision cataract surgery has been recommended for hard nucleus cataracts despite advancements in phacoemulsification technology [36]. However, if the lens nucleus can be safely and efficiently divided, phacoemulsification and aspiration can be performed without increasing the size of the incision. Thus, adopting the eight-chop technique for phacoemulsification cataract surgery could provide an optimal solution for many patients with hard nucleus cataracts.

## Figures and Tables

**Table 1:** Preoperative characteristics and intraoperative parameters.

Characteristics/Parameters	Grade IV	Grade IV Plus	Grade V	Total	*p*-Value
Number of eyes	46	26	9	81	
Age (years)	76.2 ± 9.0	78.0 ± 11.8	74.9 ± 6.9	76.6 ± 9.8	0.62 ^a^
Sex: Male	23 (50%)	15 (58%)	6 (66.7%)	44 (54.3%)	0.55 ^b^
Sex: Female	23 (50%)	11 (42%)	3 (33.3%)	37 (45.7%)	
Operative time (min)	9.4 ± 2.2	12.3 ± 3.9	15.6 ± 3.9	10.5 ± 3.4	<0.01 ^c^
Phaco time (s)	30.6 ± 10.9	44.2 ± 15.4	65.9 ± 22.0	38.9 ± 17.7	<0.01 ^c^
Aspiration time (s)	117.2 ± 30.6	147.8 ± 38.1	194.3 ± 50.7	135.6 ± 43.2	<0.01 ^c^
CDE	14.3 ± 4.4	22.8 ± 8.0	33.8 ± 12.9	19.2 ± 9.4	< 0.01 ^c^
Volume of fluid used (mL)	46.5 ± 12.0	58.3 ± 17.9	70.7 ± 26.3	53.0 ± 17.9	< 0.01 ^c^

Unless otherwise noted, values are expressed as means ± standard deviations or as percentages. ^a^ Unpaired *t*-test showed no significant differences between the groups. ^b^ Chi-square testing showed no significant differences between the groups. ^c^ One-way analysis of variance showed significant differences between the groups. CDE: cumulative dissipated energy.

**Table 2:** Pre- and postoperative CECD values.

	Mean CECD ± SD and % Decrease				
Time Period	Grade IV (*n* = 49)	Grade IV Plus (*n* = 30)	Grade V (*n* = 10)	Total (*n* = 89)	*p*-Value
Preoperatively	2530 ± 248	2496 ± 241	2622 ± 142	2529 ± 237	0.35 ^c^
7 weeks postoperatively	2518 ± 266 ^a^	2208 ± 562 ^b^	2318 ± 442 ^b^	2393 ± 432 ^b^	<0.01 ^d^
% Decrease	0.9 ± 13.6	22.5 ± 42.1	19.7 ± 40.3	10.4 ± 31.1	
19 weeks postoperatively	2503 ± 320 ^a^	2316 ± 458 ^b^	2361 ± 410 ^b^	2425 ± 394 ^b^	0.09 ^c^
% Decrease	0.2 ± 12.2	6.8 ± 18.2	9.6 ± 16.5	3.7 ± 15.3	

Values represent means ± standard deviations. ^a^ Paired *t*-tests showed no significant differences between the groups. ^b^ Paired *t*-tests showed significant differences between the groups. ^c^ One-way analysis of variance showed no significant differences between the groups. ^d^ One-way analysis of variance showed significant differences between the groups. CECD: corneal endothelial cell density; SD: standard deviation.

**Table 3:** Pre- and postoperative endothelial CCT, CV, and PHC.

	CCT, CV, and PHC				
Time Period	Grade IV (*n* = 30)	Grade IV Plus (*n* = 24)	Grade V (*n* = 9)	Total (*n* = 63)	*p*-Value
CCT	**Mean ± SD**				
Preoperatively	536.4 ± 35.7	541.0 ± 32.7	519.9 ± 44.5	535.8 ± 36.0	0.33 ^c^
7 weeks postoperatively	535.5 ± 31.9 ^a^	541.2 ± 42.7 ^a^	524.0 ± 37.2 ^a^	536.0 ± 36.9 ^a^	0.50 ^c^
19 weeks postoperatively	529.9 ± 30.0 ^b^	536.7 ± 31.3 ^b^	524.1 ± 45.9 ^a^	529.9 ± 30.0 ^b^	0.55 ^c^
CV	**Mean ± SD**				
Preoperatively	40.5 ± 5.5	44.9 ± 7.2	38.6 ± 4.2	41.9 ± 6.4	<0.01 ^d^
7 weeks postoperatively	39.2 ± 4.1 ^a^	43.4 ± 6.6 ^a^	38.1 ± 5.6 ^a^	40.6 ± 5.7 ^a^	<0.01 ^d^
19 weeks postoperatively	37.8 ± 4.9 ^b^	40.2 ± 5.1 ^b^	35.8 ± 5.6 ^b^	38.4 ± 5.2 ^b^	0.06 ^c^
PHC	**Mean ± SD**				
Preoperatively	43.5 ± 5.5	39.5 ± 6.5	47.1 ± 8.9	42.5 ± 6.9	<0.01 ^d^
7 weeks postoperatively	43.0 ± 6.1 ^a^	39.3 ± 6.0 ^a^	44.9 ± 8.6 ^a^	41.9 ± 6.7 ^a^	0.04 ^d^
19 weeks postoperatively	43.8 ± 5.3 ^a^	41.5 ± 7.4 ^a^	46.7 ± 8.4 ^a^	43.3 ± 6.7 ^a^	0.13 ^c^

Values represent means ± standard deviations. ^a^ Paired *t*-tests showed no significant differences between the groups. ^b^ Paired *t*-tests showed significant differences between the groups. ^c^ One-way analysis of variance showed no significant differences between the groups. ^d^ One-way analysis of variance showed significant differences between the groups. CCT: central corneal thickness; CV: variation in the size of endothelial cells; PHC: percentage of hexagonal cells; SD: standard deviation.

**Table 4:** Mean IOP (mmHg) and mean decrease (%) in the IOP (mmHg) over time.

	Mean IOP ± SD (% Decrease ± SD)				
Time Period	Grade IV (*n* = 46)	Grade IV Plus (*n* = 29)	Grade V (*n* = 9)	Total (*n* = 84)	*p*-Value
Preoperatively	14.1 ± 2.8	13.6 ± 2.9	13.3 ± 2.2	13.8 ± 2.8	0.84 ^c^
7 weeks postoperatively	11.7 ± 2.2 ^a^	11.7 ± 2.4 ^a^	11.1 ± 0.9 ^a^	11.6 ± 2.2 ^a^	0.78 ^c^
% Decrease	16.4 ± 11.9	13.3 ± 12.4	14.6 ± 15.0	15.1 ± 12.4	
19 weeks postoperatively	11.9 ± 1.9 ^a^	11.6 ± 2.3 ^a^	11.9 ± 2.0 ^b^	11.8 ± 2.0 ^a^	0.79 ^c^
% Decrease	13.9 ± 12.0	13.1 ± 15.7	8.5 ± 22.7	13.1 ± 14.6	

Values represent means ± standard deviations. ^a^ Paired *t*-tests showed significant differences between the groups. ^b^ Paired *t*-tests showed no significant differences between the groups. ^c^ One-way analysis of variance showed no significant differences between the groups. IOP: intraocular pressure; SD: standard deviation.

**Table 5:** Changes in corrected distance visual acuity over time.

	Corrected Distance Visual Acuity				
Time Period	Grade IV (*n* = 43)	Grade IV Plus (*n* = 21)	Grade V (*n* = 8)	Total (*n* = 72)	*p*-Value
Preoperatively	0.50 ± 0.59	0.88 ± 0.83	0.89 ± 0.66	0.65 ± 0.69	0.07 ^b^
7 weeks postoperatively	−0.020 ± 0.17 ^a^	−0.011 ± 0.058 ^a^	0.0079 ± 0.050 ^a^	−0.014 ± 0.13 ^a^	0.86 ^b^
19 weeks postoperatively	−0.028 ± 0.17 ^a^	−0.035 ± 0.050 ^a^	−0.014 ± 0.043 ^a^	−0.029 ± 0.13 ^a^	0.93 ^b^

Values represent means ± standard deviations. ^a^ Paired *t*-tests showed significant differences between the groups. ^b^ One-way analysis of variance showed no significant differences between the groups.

## Data Availability

The data presented in this study are available on request from the corresponding author due to privacy and ethical restrictions.

## Abbreviations

The following abbreviations are used in this manuscript:

CECD	Corneal endothelial cell density
CDE	Cumulative dissipated energy
IOP	Intraocular pressure
IOL	Intraocular lens
CDVA	Corrected distance visual acuity
CCT	Central corneal thickness
PHC	Percentage of hexagonal cells
CV	Variation in the size of the endothelial cells
SD	Standard deviation
